# Men’s Health & Sexual Medicine and the Litigious Patient: A Review of Malpractice Cases

**DOI:** 10.5152/tud.2025.24163

**Published:** 2025-04-04

**Authors:** Sandeep Sai Voleti, Summer Ghaith, Christopher Warren, Nahid Punjani

**Affiliations:** 1 Department of Urology, Mayo Clinic, Arizona, United States; 2 Mayo Clinic Alix School of Medicine, Arizona, United States

**Keywords:** Malpractice, patient centered care, urology

## Abstract

**Objective:**

Recent literature suggests growing rates of malpractice claims against urologists. These cases provide insight into errors that may lead to litigation. We aim to analyze malpractice suits related to men’s health and highlight clinical presentations and legal outcomes.

**Methods:**

Per STROBE guidelines, we searched the publicly available Thomson Reuters Westlaw legal database for “jury verdicts and settlements” from January 1970 to August 2023 to identify medical malpractice cases in Urology. Patient demographics, clinical presentation, alleged error, and legal outcomes were abstracted during a full case review by an independent screener. Cases related to men’s sexual health or fertility that named a urologist as the defendant were fully analyzed.

**Results:**

A total of 553 urology cases were extracted, with 23 men’s health related cases subsequently analyzed. The most common conditions associated with litigation were penile prosthesis (39%), epidydimal pathology (13%), and varicocele (13%). The most common allegations were misdiagnosis (26%), surgical complication (22%), lack of informed consent (22%), and violation of standard of care (22%). Of the 23 cases, 57% ruled “no liability” in favor of the urologist and 39% ruled in favor of the plaintiff with a median award of $335,000 (IQR = 100 ,000-450, with 000). Among cases ruled as negligent, performing the incorrect procedure, surgical complications, and violating the standard of care were prominent allegations.

**Conclusions:**

This study characterizes malpractice cases related to men’s sexual health naming urologists as defendants. Obtaining comprehensive informed consent, following national guidelines, and responding appropriately to surgical complications may minimize likelihood of litigation and maximize patient outcomes.

**Main Points**

- There are increasing rates of malpractice suits against physicians nationally, and understanding malpractice cases in men’s health will allow us to better care for our patients.
- The most common alleged errors by patients in men’s health malpractice suits are missed/delayed/misdiagnosis, surgical complications, and lack of informed consent.
- The most common pathologies in men’s health associated with malpractice litigation are penile prosthesis, epididymal pathology, and varicocele.

## Introduction

Recent literature highlights national trends of persistent risk of malpractice claims against urologists (63% report being named in a mean of 2.1 lawsuits).^1^,^2^ These cases may provide insight with respect to common medical errors contributing to litigation. Prior studies have highlighted misdiagnosis and lack of informed consent as common alleged errors in successful lawsuits in the setting of assisted reproductive technologies.^3^ Given the significant emotional investment associated with men’s health, understanding litigation related to men’s sexual and reproductive health is critical to enhance current standards of care. We aim to characterize men’s sexual health and fertility-related malpractice lawsuits to provide insight into patient characteristics and clinical presentations, as well as describe the final outcomes of such cases.

## Material and Methods

This study was exempt from Ethics Committee Approval as it utilized publicly available data. Per the STROBE guidelines for observational studies, the publicly available Thomson Reuters Westlaw legal database was searched for “jury verdicts and settlements” from January 1970 to August 2023 to identify medical malpractice cases using search terms “urology + malpractice, urology + malpractice + vasectomy, urology + malpractice + circumcision, and urology + malpractice + penile prosthesis/penile implant/IPP.” An independent screener queried each malpractice case for patient demographics, clinical presentation, alleged error, and legal outcomes prior to comprehensive case review. Cases related to men’s sexual health/fertility and that named a Urologist as a defendant were fully analyzed. Descriptive statistics were utilized to highlight the current landscape of sexual health malpractice cases.

## Results

The initial query of the Westlaw database yielded 553 cases. After removing duplicates and cases not explicitly related to men’s sexual health and fertility and not naming a Urologist as a defendant, 23 (4%) cases remained to be fully analyzed. The most common conditions associated with litigation were penile prosthesis (39%), epidydimal pathology (13%), and varicocele (13%) (Table 1).

The most common alleged complaints were missed/delayed/misdiagnosis (26%), surgical complication (22%), lack of informed consent (22%), and violation of standard of care (22%) (Table 2). Of the 23 cases reviewed, 9 (39%) resulted in negligence, with the median award amount being $335 000 (IQR = 100 000-450 000). One case resulted in a settlement with a monetary award of $316, 338. The remaining cases resulted in no liability. Amongst cases that resulted in a verdict of negligence, performing the incorrect procedure, surgical complications, and violation of standard of care were prominent allegations.

## Discussion

Overall, we identified 23 malpractice cases related to male sexual medicine and reproduction. The most frequently alleged errors were missed, delayed, and misdiagnosis, and 39% of cases ruled negligence on the part of the provider.

Patients receiving a penile prosthesis comprised nine of the total cases, with three cases ruling negligence. Penile prosthesis remains the final definitive treatment of erectile dysfunction, and given the nature of the procedure, complications may foster greater dissatisfaction compared to other procedures.

Surprisingly, only a single vasectomy case was noticed in this sample. A prior study had identified a notable number of malpractice cases related to vasectomy, with negligence in postoperative care and negligent surgical performance being noted as the top two alleged errors.^4^ This discrepancy may be due to the inclusion of only malpractice cases that named urologists as defendants, or to differences in the search terms used.

The most frequent alleged errors identified in this study highlight grievances that likely lead a patient to be dissatisfied with their care and to pursue litigation. Missed, delayed, and misdiagnosis, being the most common alleged errors, highlight the value patients place on comprehensive and accurate diagnostic measures. Examples of clinical scenarios that led to these allegations include misdiagnosing a testicular torsion as epididymitis necessitating subsequent orchiectomy, delayed diagnosis of infection of a penile prosthesis (leading to gangrene), and allowing for a period of five months to pass before sharing ultrasound results with a patient showing that they have a concerning testicular mass, resulting in delayed treatment. Lack of informed consent and violation of standard of care were also commonly alleged errors, highlighting key areas in which physicians can improve their practice and patient satisfaction. Notably, one case outlined a situation where a provider consented a patient for an intraoperative frozen section biopsy of the testicle, but instead performed an orchiectomy without consenting the patient for it prior. Two separate cases alleged that providers did not adequately counsel patients regarding symptoms/complications following varicocelectomy, including swelling, discomfort, and injury to the testicular artery. Performing the incorrect procedure and violation of standard of care were common allegations among cases that ruled negligence, highlighting easily correctable practices. The aforementioned cases showcase clear areas in which it is crucial that providers comprehensively counsel their patients on treatment options as well as procedural risks and benefits. In current practice, pre-operative marking and surgical time-out have been shown to minimize but not completely eradicate risks of incorrect procedures.^5^,^6^ However, surgical complications were also common allegations among successful lawsuits by patients. Examples of surgical complications outlined in these cases include orchitis with hemorrhage and abscess formation following hydrocelectomy as well as mis-sizing of a penile prosthesis leading to poor perfusion and gangrene. While surgical complications are inevitable, efforts to reduce complications and identify and address them early may decrease litigation related to surgical complications.^7^

While this study provides critical insight into urologic litigation, it is limited by the use of the Westlaw database, which is considered comprehensive but not exhaustive in records of malpractice suits. Further, this study solely includes malpractice cases filed in the United States. Medicolegal environments vary significantly between different countries; therefore, this study serves to provide further insight into how providers can enhance care to their patients with improved satisfaction but does not provide specific legal-based recommendations. However, it is notable that literature exploring urology-related malpractice cases in other countries has identified similar patient grievances. One study focusing on malpractice cases in South Korea identified a notable portion of cases alleging violations of informed consent and duty of care (including surgical complications), with examples including injury to the vasal artery during vasectomy, improper treatment of glans necrosis, and failure to provide adequate counseling regarding possible complications following surgery.^1^ Procedural skill errors and lack of informed consent were also cited as the most frequently alleged complaints in another study focusing on urology-related malpractice suits in Türkiye, suggesting that, while legal policies may vary from country to country, foundational principles of patient care are universally expected from providers by patients.^8^

This study highlights the need for further investigation into the factors that most influence a patient’s likelihood of pursuing litigation. Prospective data collection in this domain may provide further insight to help improve urology practice, enhance outcomes, and reduce future litigation.

## Conclusions

This review characterizes malpractice cases related to men’s sexual health and fertility. It highlights the conditions that are most likely to lead to litigation, as well as allegations that are more likely to result in successful lawsuits. Obtaining comprehensive informed consent, following the standards of care set by national guidelines, and responding appropriately to surgical complications may minimize likelihood of litigation.

## Figures and Tables

**Table 1.** Frequency of Malpractice Lawsuits Regarding Men’s Sexual Health and Fertility by Clinical Pathology

Pathology	Number of Malpractice Cases
Peyronie’s disease	0
Scrotal sebaceous cyst	1
Vasectomy	1
Hydrocele	2
Testicular mass	2
Testicular torsion	2
Epidydimal pathology	3
Varicocele	3
Penile prosthesis	9

**Table 2.** Summary of Malpractice Cases Regarding Men’s Sexual Health and Fertility Care

Case Number	State	Clinical Picture	Alleged Error/Complaint	Legal Outcome	$ Amount (If Pertinent)
1	New York	Epidermoid cyst	Misdiagnosis/failure to diagnose	No liability	–
2	Florida	Epidydimal cyst	Incorrect procedure performed; violation of standard of care	Negligence	$300 000
3	Pennsylvania	Epididymitis	Incorrect procedure performed; misdiagnosis; lack of informed consent	Negligence	$50 000
4	California	Hydrocele	Violation of standard of care	No liability	–
5	Texas	Hydrocele	Violation of standard of care; surgical complication	No liability	–
6	Oregon	Penile prosthesis	Surgical complication	Negligence	$1 000 000
7	New York	Penile prosthesis	Surgical complication; delayed diagnosis	Negligence	$6 500 000
8	Florida	Penile prosthesis	Surgical complication; violation of standard of care	Negligence	$100 000
9	New Jersey	Penile prosthesis	Failure to perform surgery properly	No liability	–
10	Florida	Penile prosthesis	Failure to perform surgery properly	No liability	–
11	Maryland	Penile prosthesis	Failure to perform surgery properly	No liability	–
12	Florida	Penile prosthesis	Failure to perform surgery properly; misdiagnosis	No liability	–
13	Michigan	Penile prosthesis	Surgical complication	No liability	–
14	Illinois	Penile prosthesis	Surgical complication; lack of informed consent	No liability	–
15	Nevada	Scrotal sebaceous cyst	Incorrect procedure performed	Negligence	$63, 881
16	Pennsylvania	Testicular mass	Delayed diagnosis	Negligence	$374, 500
17	Florida	Testicular mass	Lack of informed consent; incorrect procedure performed	Negligence	$450, 000
18	Pennsylvania	Testicular torsion	Misdiagnosis; delayed diagnosis	No liability	–
19	Florida	Testicular torsion	Missed diagnosis; delayed diagnosis	No liability	–
20	New York	Varicocele	Violation of standard of care; lack of informed consent; surgical complication	Negligence	$335, 000
21	Pennsylvania	Varicocele	Lack of informed consent; failure to perform surgery properly; surgical complication	No liability	–
22	New Jersey	Varicocele	Lack of informed consent; unnecessary intervention	No liability	–
23	California	Vasectomy	Inaccurate portrayal of results; negligence	Settlement	$316, 338

## Data Availability

The data that support the findings of this study are available in the Thomas Westlaw-Reuters database.
